# Time-Resolved Infrared Spectroscopy Reveals the pH-Independence of the First Electron Transfer Step in the [FeFe] Hydrogenase Catalytic Cycle

**DOI:** 10.1021/acs.jpclett.2c01467

**Published:** 2022-06-23

**Authors:** Monica L. K. Sanchez, Seth Wiley, Edward Reijerse, Wolfgang Lubitz, James A. Birrell, R. Brian Dyer

**Affiliations:** † Department of Chemistry and Biochemistry, Montana State University, Bozeman, Montana 59717, United States; ‡ Department of Chemistry, Emory University, Atlanta, Georgia 30030, United States; § Max Planck Institute for Chemical Energy Conversion, Stiftstraße 34-36, 45470 Mülheim an der Ruhr, Germany

## Abstract

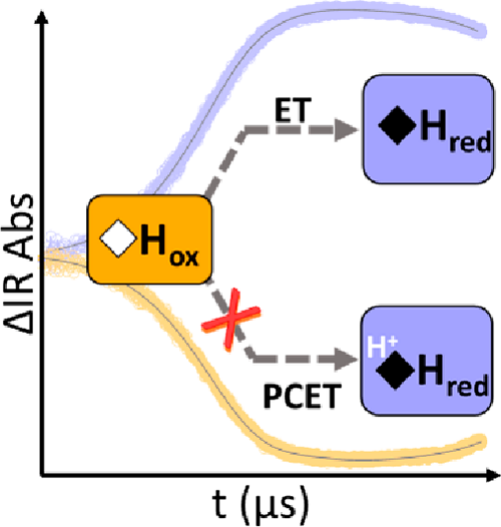

[FeFe] hydrogenases are highly active catalysts for hydrogen conversion. Their active site has two components: a [4Fe−4S] electron relay covalently attached to the H_2_ binding site and a diiron cluster ligated by CO, CN^–^, and 2-azapropane-1,3-dithiolate (ADT) ligands. Reduction of the [4Fe−4S] site was proposed to be coupled with protonation of one of its cysteine ligands. Here, we used time-resolved infrared (TRIR) spectroscopy on the [FeFe] hydrogenase from *Chlamydomonas reinhardtii* (*Cr*HydA1) containing a propane-1,3-dithiolate (PDT) ligand instead of the native ADT ligand. The PDT modification does not affect the electron transfer step to [4Fe−4S]_H_ but prevents the enzyme from proceeding further through the catalytic cycle. We show that the rate of the first electron transfer step is independent of the pH, supporting a simple electron transfer rather than a proton-coupled event. These results have important implications for our understanding of the catalytic mechanism of [FeFe] hydrogenases and highlight the utility of TRIR.

[FeFe] hydrogenases are highly active hydrogen producing enzymes requiring only Fe as the redox center in their active site.^1−4^ The properties of the Fe core are tuned by the ligand coordination and the protein environment to create a highly efficient catalytic center. The active site H-cluster is composed of a diiron subcluster ([2Fe]_H_) covalently bound to the cysteine thiol of a [4Fe−4S] subcluster ([4Fe−4S]_H_).^5−8^ The Fe atoms in [2Fe]_H_ are coordinated by terminal CO and CN^–^ ligands (one on each iron). A third CO and a 2-azapropane-1,3-dithiolate (ADT) ligand bridge the two iron ions^9−11^ (Figure 1A). Hydrogen is thought to bind to the active oxidized (H_ox_) state between the distal Fe (that farthest from [4Fe−4S]_H_) and the nitrogen of the ADT ligand.^12^ Together, the nitrogen base and the low valent Lewis acidic Fe are thought to form a frustrated Lewis pair, heterolytically splitting H_2_.^13^ Substitution of this nitrogen with carbon leads to an essentially inactive enzyme (see Supplementary Discussion).^10,14^ Clearly, the nitrogen base is critical for efficient catalysis.^15^

**Figure 1:**
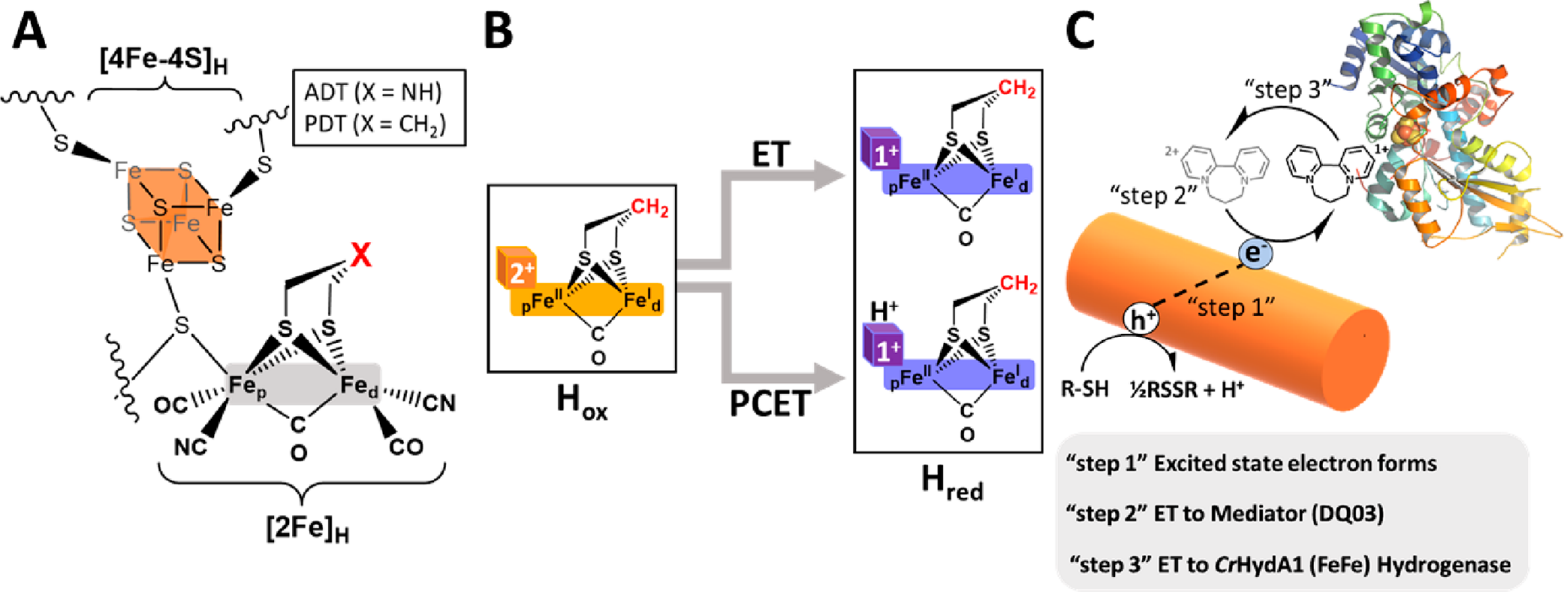
Description of the reaction pathways and experimental approach. (A) Illustration of the H-cluster with the bridgehead atom is highlighted as a red X and can be NH in the ADT variant and CH_2_ in the PDT variant. (B) Schematic describing the proposed pathways from H_ox_ to H_red_ (Hred′). (C) Schematic of the overall ET pathway from photosensitizer to mediator to enzyme catalyst. R-SH = the sacrificial electron donor (SED), mercaptopropionic acid (MPA), and RSSR = oxidized SED 3,3′-dithiodipropionic acid.

It has been suggested that [4Fe−4S]_H_ can also be protonated on one or more of its cysteine thiolate groups (see Supplementary Discussion).^16,17^ This protonation step has been suggested to prevent the formation of inactive bridging hydride bound forms of [2Fe]_H_.^18,19^ However, both the protonation of [4Fe−4S]_H_ and the formation of bridging hydrides have recently been called into question.^20−23^ However, these thermodynamic studies cannot completely exclude that protonation of [4Fe−4S]_H_ does indeed occur, but with a p*K*_a_ value outside of the range where the enzyme is stable, and so it cannot be observed. If the electron transfer step is coupled to proton transfer, the rate of proton transfer observed in kinetics experiments should be sensitive to proton concentration. Further exploration is needed to resolve the controversy surrounding the proton-coupling of the first ET step. Kinetics measurements, particularly time-resolved infrared (TRIR) spectroscopy, can give insight into whether the first ET step is truly a PCET event or pure ET.^24^ Insight can be gleaned by monitoring the formation of the first one-electron reduced state population under different pH conditions. Here, we use CdSe/CdS nanorods (NRs) to photoreduce a low potential redox mediator (DQ03), which in turn transfers electrons to [FeFe] hydrogenase on a time scale faster than enzyme turnover, as described previously^25−27^ (Figure 1C).

For the time-resolved measurements described in this study, we used a variant of an [FeFe] hydrogenase from the organism *Chlamydomonas reinhardtii* (*Cr*HydA1). In this variant, the natural ADT ligand, which contains a nitrogen as the bridgehead atom, is exchanged for a propane-1,3-dithiolate (PDT) ligand (Figure 1A). The PDT substitution presumably prevents protonation of the bridging ligand in [2Fe]_H_, which in turn prevents electron transfer from [4Fe−4S]_H_ to [2Fe]_H_, trapping the H-cluster in a one-electron reduced state, effectively abolishing catalysis.^10,28,29^ This effect allows us to clearly monitor the first reduction of [4Fe−4S]_H_ going from the oxidized (H_ox_) state to the one-electron reduced (H_red_) state without further conversion into other catalytic states (Figure 1B). Otherwise, the spectral properties of the ADT and PDT variants in the H_ox_ and H_red_ states are very similar.^23,29,30^ It should be noted that elsewhere in the literature the one-electron reduced state in which [4Fe−4S]_H_ is reduced has been named Hred′ and is assigned as having a protonated Fe-ligating cysteine.^16,17^

Three scenarios can be envisaged: reduction of H_ox_ to H_red_ is (1) pure ET, (2) PCET where ET is slow compared with PT, and (3) PCET where ET is more rapid than or equal to PT. If the formation of H_red_ is ET only or PT is extremely rapid, then no difference in the rate of formation will be observed at different pH values. If, however, this first ET is coupled to (partially) rate-limiting PT then the rate of formation of the H_red_ (Hred′) state will display pH-dependent behavior.^24^ This fine-tuned potential jump approach is ideal for studying these PCET and ET steps^25,27,31^ and means that any differences in the rate of ET with pH are due to PCET in the enzyme. Unfortunately, a lack of pH-dependent behavior could also mean extremely rapid (subμs) PT. However, if this is the case, the initial PCET to [4Fe−4S]_H_ cannot explain the pH-dependent activity profiles of [FeFe] hydrogenases as is occasionally proposed.^16−19^ Instead, a slower, rate-limiting proton dependent step (e.g. backfilling of a deprotonated amino acid) should be involved. Regardless, it is important to first explore whether the conversion of H_ox_ to H_red_ is pH dependent.

We first performed an equilibrium light-titration experiment with the PDT form of *Cr*HydA1. The sample was prepared with NRs and a mediator at pH 8.2 (phosphate buffer) and exposed to increasing light intensity (at 405 nm) while monitoring the active site of the enzyme by Fourier transform infrared (FTIR) spectroscopy (Figure S3). The PDT sample displayed a decrease in the intensity at 1942 cm^–1^ from the H_ox_ population, consistent with reduction of the H-cluster.^29^ An increase of the intensity at 1935 cm^–1^ due to formation of the H_red_ state was also observed.^29^ The different intensities of the H_ox_ and H_red_ peaks in the difference spectrum is due to different peak widths and extinction coefficients. The isosbestic point is a clear indication of two-state behavior, indicating direct conversion of H_ox_ to H_red_.

Next, we sought to assess the pH dependence of the kinetics of the first ET step. We performed time-resolved measurements monitoring the rate of formation of H_red_ at pH 6.5 and 8.2 by monitoring at frequencies associated with H_ox_ (1942 cm^–1^) and H_red_ (1935 cm^–1^) (Figure 2A). The population of the reduced mediator was also monitored with time-resolved visible (TRVis) methods using the absorbance at 785 nm (Figure S5). Previous studies by our groups established a relationship between pH and the efficiency of the mediator reduction by NRs.^25^ As the pH becomes more alkaline, hole transfer from the rod to the sacrificial electron donor becomes faster, resulting in a net increase in ET efficiency to the mediator. These factors culminate in a larger, more negative solution potential jump, which can influence the rate of ET to the enzyme.^25^ To compensate for the pH dependent efficiency, we have carefully attenuated the excitation source to obtain consistent potential jumps at each pH value. Consistent potential jumps were confirmed by TRVis studies monitoring the population of the reduced mediator in solution. The concentration of the DQ03 radical generated was kept within a range of 220 μM to 270 μM (based on the absorbance at 785 nm) corresponding to solution potential jumps between −425 mV and −435 mV vs SHE.

**Figure 2:**
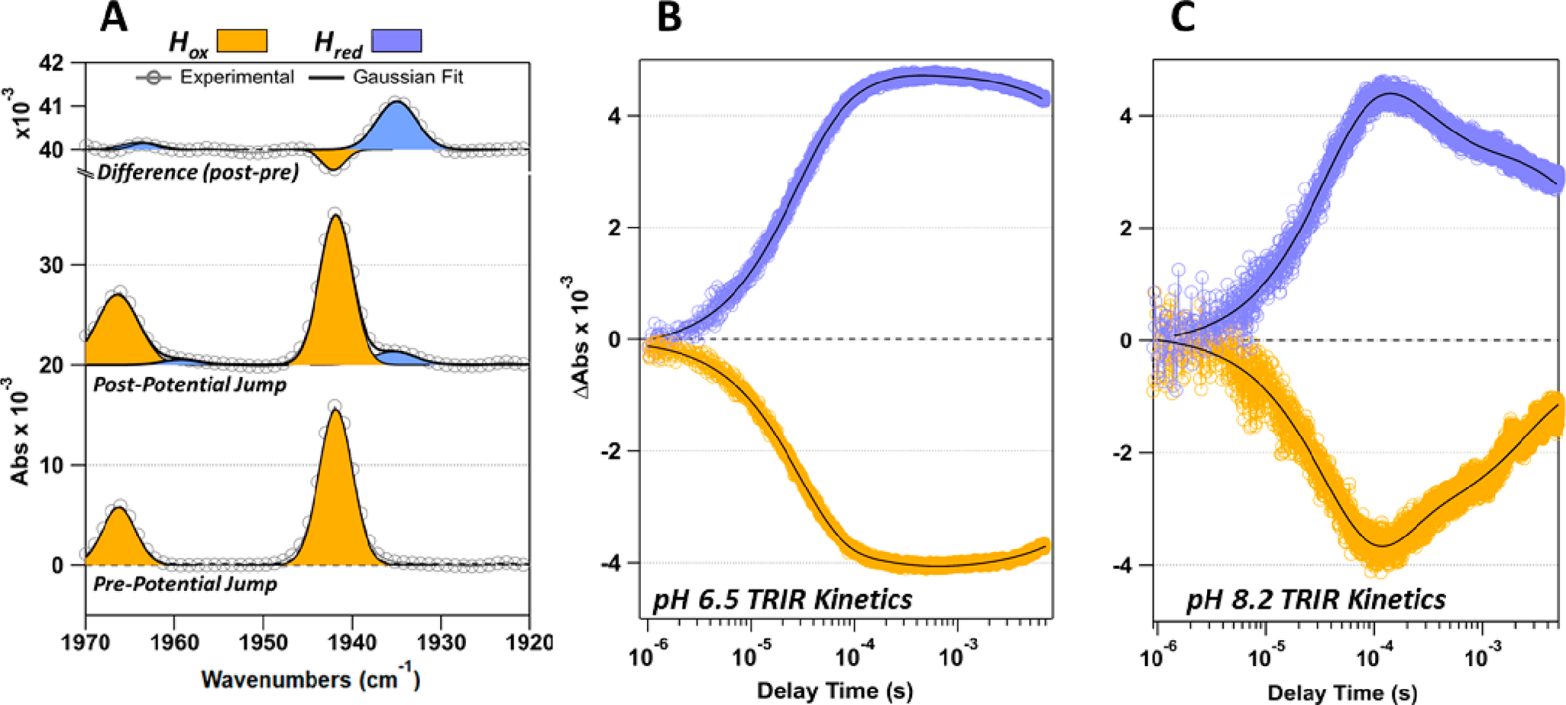
Summary of TRIR results monitoring H_red_ and H_ox_ at 1935 and 1942 cm^–1^, respectively. (A) Representative FTIR before and after potential jump kinetics measurements. (B) TRIR of pH 6.5 sample monitoring H_ox_ and H_red_ states. (C) TRIR of pH 8.2 sample monitoring H_ox_ and H_red_ states. Color scheme is as follows: gold = H_ox_, light blue = H_red_, black = 3-exponential fit.

The radical consumption rates measured at three different laser intensities (2 mW, 4 mW, and 6 mW) were essentially identical (Figure S6), indicating that the radical consumption rate was not diffusion limited. Furthermore, the rates of radical consumption, H_ox_ decrease, and H_red_ increase were all the same, indicating that electron transfer from the mediator to the active site of the enzyme was rate limiting.

For both samples, FTIR spectra were recorded before and after the TRIR experiments to monitor the formation (or depletion) of the H_ox_ and H_red_ populations present in each sample (Figure S4). A representative FTIR spectrum from the pH 6.5 sample, taken before the time-resolved measurements, reveals a sample completely in the H_ox_ state (Figure 2A). Spectra recorded after the time-resolved IR experiment show the appearance of a population of H_red_. Difference spectra, generated by subtracting the prepotential jump (pre-PJ) spectra from the post-PJ spectra, highlight the changes in population which occurred during the PJ experiment.

Figure 2, panels B and C, display TRIR kinetics traces for the pH 6.5 and 8.2 samples. Individual kinetics traces (25–30 traces) are collected at each frequency for the sample as well as a reference. Each data set is then averaged, and the reference signal is subtracted from the sample, leaving a change in absorbance (ΔA) related exclusively to the change in population of each state.

The decrease in H_ox_ (1942 cm^–1^) matches the increase in H_red_ (1935 cm^–1^; ΔAbs = 4 × 10^–3^) and occurs on a similar time scale for both pH 6.5 (Figure 2B) and 8.2 (Figure 2C). Fitting the data with a multiexponential function (Figures S7–S10) shows that, at pH 6.5, H_ox_ decays with a lifetime of 28 μs, while H_red_ forms with a lifetime of 25 μs. At pH 8.2, H_ox_ decays with a lifetime of 30 μs, while H_red_ forms with a lifetime of 29 μs. The rate of mediator decay, H_ox_ decay, and H_red_ formation are all the same within the error of the measurement (Figures S6–S10). Furthermore, the behavior at pH 6.5 and 8.2 is identical despite the difference in pH of 1.7 units (corresponding to a 50-fold difference in H^+^ concentration). These values are similar to those reported for the formation of H_red_ from H_ox_ in the native ADT-variant in our previous study.^26^

Unexpectedly, the population of H_red_ appears to decay on longer time scales. The H_red_ decay appears to be much faster at pH 8.2, where the decay in H_red_ occurs with a lifetime of 840 μs and the reformation of H_ox_ occurs with a lifetime of 1.2 ms, compared to pH 6.5, where the decay in H_red_ occurs with a lifetime of 43 ms and the reformation of H_ox_ occurs with a lifetime of 46 ms. These latter values indicate that over time the population of H_red_ is not stable at slightly alkaline pH values. H^+^ reduction by the enzyme is not likely to be the explanation as this should be faster at low pH not high pH. Instead, reoxidation of the enzyme by 3,3′-dithiodipropionic acid (RSSR in Figure 1C), the product of sacrificial oxidation of 3-mercaptopropanoic acid (RSH in Figure 1C) by the NR, could be accelerated at high pH due to favorable charge interactions between the deprotonated carboxylate groups of RSSR and the positively charged region around [4Fe−4S]_H_ in *Cr*HydA1.^32^ The discrepancies in the signal of H_ox_ and H_red_ at longer time points, especially at pH 8.2, are attributed to imperfect removal of the contribution from sample heating (see Figure S11 and details in ref (26)).

In summary, we find that the reduction of the oxidized (H_ox_) state of [FeFe] hydrogenase to the one-electron reduced state has a pH-independent rate constant. A pH-independent rate constant is not consistent with a PCET event where protonation happens on comparable time-scales to electron transfer but is more consistent with a simple ET event. These results are in agreement with the pH-independent redox potential of the [4Fe−4S]_H_ subcluster of the H-cluster determined in the absence of NaDT.^21^ We conclude that catalytically important protonation occurs at the nitrogen of the ADT bridge, leading to proton-coupled electronic rearrangement (PCER) of the H-cluster to give a protonated, one-electron reduced state (H_red_H^+^) that is ready to accept an additional electron on [4Fe−4S]_H_. This PCER process was originally proposed based on the fact that the H_red_ and H_red_H^+^ states vary their populations with pH, with H_red_ being prevalent at high pH and H_red_H^+^ being prevalent at low pH. Recently, the same finding was interpreted by Laun et al. as evidence that both sites can be protonated but that the proton shifts between the two sites in a pH-dependent fashion.^33^ This later interpretation is not chemically intuitive as the H_red_ and H_red_H^+^ states would be tautomers. Our current results provide even further evidence against this idea as the rates of H_red_ formation are also pH-independent. Overall, these results highlight the utility of using time-resolved spectroscopy approaches for studying enzyme mechanisms, especially such active catalysts as [FeFe] hydrogenases.

## Supporting Information Available

Supplementary discussion, materials preparation and characterization, FTIR spectra, and data analysis (PDF)

## Abbreviations

ADT: 2-azapropane-1,3-dithiolate
PDT: propane-1,3-dithiolate
TRIR: time-resolved infrared
TRVis: time-resolved visible
PCET: proton-coupled electron transfer
ET: electron transfer
NR(s): nanorod(s)
*Cr*HydA1: *Chlamydomonas reinhardtii* hydrogenase
PCER: proton coupled electronic rearrangement
